# Mid-Term Outcome of Mechanical Pulmonary Valve Prostheses: The Importance of Anticoagulation

**DOI:** 10.15171/jcvtr.2014.005

**Published:** 2014-09-30

**Authors:** Anita Sadeghpour, Majid Kyavar, Bahareh Javani, Hooman Bakhshandeh, Majid Maleki, Zahra Khajali, Lakshman Subrahmanyan

**Affiliations:** ^1^Rajaie Cardiovascular, Medical and Research Center, Iran University of Medical Sciences, Tehran, Iran; ^2^Section of Cardiology, Department of Medicine, Yale University School of Medicine, New Haven, Connecticut, USA

**Keywords:** Plumonary Valve Replacement, Mechanical Prosthese, Anticogulation, Warfarin

## Abstract

*Introduction:* Pulmonary valve replacement (PVR) is being performed more commonly late after the correction of tetralogy of Fallot. Most valves are replaced with an allograft or xenograft, although reoperations are a common theme. Mechanical prostheses have a less favorable reputation due to the necessity of lifelong anticoagulation therapy and higher risk of thrombosis, but they are also less likely to require reoperation. There is a paucity of data on the use of prosthetic valves in the pulmonary position. We report the midterm outcomes of 38 cases of PVR with mechanical prostheses.

*Methods:* One hundred twenty two patients who underwent PVR were studied. Thirty-eight patients, mean age 25 ± 8.4 years underwent PVR with mechanical prostheses based on the right ventricular function and the preferences of the patients and physicians. Median age of prosthesis was 1 year (range 3 months to 5 years).

*Results: * Seven (18%) patients had malfunctioning pulmonary prostheses and two patients underwent redo PVR. Mean International Normalized Ratio (INR) in these seven patients was 2.1±0.8. Fibrinolytic therapy was tried and five of them responded to it well. There was no significant association between the severity of right ventricular dysfunction, patient’s age, prostheses valve size and age of the prosthesis in the patients with prosthesis malfunction.

*Conclusion: * PVR with mechanical prostheses can be performed with promising midterm outcomes. Thrombosis on mechanical pulmonary valve prostheses remains a serious complication, but most prosthesis malfunction respond to fibrinolytic therapy, underscoring the need for adequate anticoagulation therapy.

## Introduction

There is currently a dearth of information in the medical literature on the use of prosthetic valves in the pulmonary position. Most of the available data come from small sets of patients, mostly in the pediatric population with an underlying congenital heart disease.^1^

Pulmonary regurgitation is unusual as an isolated congenital defect, but it is an almost unavoidable result of either surgical or balloon valvuloplasty of valvular pulmonic stenosis or surgical repair of Tetralogy of Fallot (TOF),^2^ which constitutes one of the most common forms of cyanotic congenital heart disease. Primary intracardiac repair for TOF canbe performed with excellent short-term and long-term results.^3-5^ Late prognosis after TOF repair is excellent, with a 35-year survival rate of 85%.^6^ Although long-term results are good, pulmonary regurgitation is very common after TOF repair. Severe chronic pulmonary regurgitation eventually leads to symptomatic right ventricle (RV) dilation and dysfunction as well as increased risk of arrhythmias and sudden death,^7-10^ accounting for the increasing numberof pulmonary valve replacement (PVR) cases in patients with repaired TOF.

Most surgeons tend to replace the pulmonary valve with an allograftor xenograft, and there have been reports of favorable medium-term follow-up.^11-13^ Nonetheless, both of these tissue valves are liable to deteriorate overtime, necessitating (potentiallymultiple) reoperations with concomitant morbidityand mortality.^14,15^

This study evaluates the medium-term outcome of PVR with mechanical pulmonary valve prostheses in patients with severe chronic pulmonary regurgitation and underlying congenital heart disease and estimates the risk of pulmonary thrombosis in these patients.

## Materials and methods

### Patient population

Between March 2003 and July 2008, 122 patients with a history of TOF repair (82%) or pulmonary valvotomy (18%) underwent PVR in our center. The decision for PVR was made by a team, consisting of cardiologists and cardiac surgeons, based on a patient’s symptoms and the current criteria for PVR. Thirty-eight patients underwent PVR with bileaflet mechanical prostheses (St. JudeMedical, St. Paul, MN). All the patients were operated on througha median sternotomy with cardiopulmonary bypass. The choice of valve type was dependent on the RV function as well as the preferences of the patients and physicians. The median age of the prostheses (time interval between PVR and echocardiographic examination) was one year, and the age of the prosthesis was between 0.5 and 5 years in 31 (82%) patients. Patients with RV to pulmonary artery conduit and Ross operation were excluded.

At follow up, all the patients underwent a complete two-dimensional (2D) and Doppler study by Vivid 3 Cardiovascular Ultrasound System (GE Medical Systems, Milwaukee, Wisconsin, USA) and the following measurements were obtained: Right ventricular size, Right ventricular function (graded according to guidelines),^16^ pulmonary valve prosthesis peak pressure gradient, mean pressure gradient, peak velocity, Right Ventricular Outflow Tract (RVOT) velocity time integral (VTI, 1cm above the pulmonary valve in this study), RVOT/PV VTI ratio, and pulmonary insufficiency by color flow imaging.

Color flow mapping of the pulmonary prostheses and branch pulmonary arteries was utilized to grade the severity of pulmonary regurgitation. Any regurgitation graded at least moderate was defined as significant. The peak systolic pressure gradient across the mechanical pulmonary prostheses was estimated by continuous wave Doppler echocardiography, using the modified Bernoulli equation, and graded as moderately stenotic (peak gradient= 36–64 mmHg) or severely stenotic (peak gradient >64 mmHg), based on the latest guideline for the assessment of valve stenosis.^17^

All the cases of prosthesis malfunction suspected based on the transthoracic echocardiography were confirmed by cinefluoroscopy. Regurgitation or stenosis greater than moderate was defined as abnormal. In all the patients with prosthesis malfunction, at least one of the leaflets of the valve was immobilized in fluoroscopy.

All the patients were anticoagulated with warfarin and aspirin. The coagulation states of the patients were assessed by measuring the INR at the time of examination and the previous six months.

### Statistical Analysis

The data are described as mean ± standard deviation for the interval and count (percent) for the categorical variables. Fitness of interval data to normal distribution was investigated using the one-sample Kolmogorov-Smirnov test. The Student *t*-test, Mann-Whitney U-test, Pearson Chi-square test, or Fisher exact test were used for the statistical analyses. P<0.05 were considered statistically significant. Logistic regression models were applied for multivariable analysis, and the life-table method was employed for survival analysis. The statistical analyses were carried out using SPSS 17 for Windows (SPSS Inc., Chicago, Illinois).

## Results

### Background Data

Thirty-eight patients with bileaflet mechanical pulmonary valve prostheses were enrolled. The mean of age was 25 ± 8.4 years (range, 14 to 60 years) and the female/male ratio was 13/25. The median age of the prostheses (time interval between PVR and echocardiographic examination) was one year (range, 3 months to 5 years), and the age of the prosthesis in 31 (82%**)** patients was between 0.5 and 5 years. The patients’ clinical data are presented in Table 1.

**Table 1:** Clinical findings in patients with mechanical pulmonary valve prosthesis (n=38)

	**Count (%)**
Symptoms	6 (16%)
Underlying Disease	
TOF	31 (82%)
PS	7 (18%)
Age of Prosthesis	
<6 months	7 (18%)
6 months–5 years	31 (82%)
Prosthesis Malfunction	7 (18%)

Most patients [31 (81%)] had a preserved or mildly reduced left ventricular function. Normal RV size was found in only 4 (11%) patients (Table 2). Most patients [27 (71 %)] had moderate or severe RV enlargement. All patients had RV dysfunction (Table 3). Seven (18%) patients had mechanical prosthesis malfunction. Prosthesis malfunction was defined based on the Doppler echocardiography and cinefluoroscopy. Regurgitation or stenosis greater than moderate were defined abnormal so cinefluoroscopy was done for all patients with abnormal functioning prosthesis by echocardiography. Malfunctioning prosthesis in cinefluoroscopy had obstructing and immobilizing one of the leaflets of the valve with or without restricted motion of another one. Interestingly the leaflets were commonly fixed in the semi-open position. The mean of INR value in the patients with prosthesis malfunction at the time of admission was 2.1. All the patients with prosthesis malfunction underwent thrombolytic therapy. Five patients responded to the thrombolytic therapy (streptokinase for 24 to 48 hours) with significant improvement in regurgitation or stenosis in echocardiography and normal leaflet motion in cinefluoroscopy. Two patients underwent redo PVR surgery, during which tissue ingrowth with superimposition of thrombi was detected.

**Table 2:** Echocardiographic parameters in patients with mechanical pulmonary valve prosthesis (n=38)

	**Count (%)**
Prosthesis Insufficiency	
No	28 (74%)
Mild	2 (5%)
Moderate	6 (16%)
Moderate to Severe	2 (5%)
Increased Peak Pressure Gradient	
Mild (<36 mmHg)	29 (76%)
Moderate (36–64 mmHg)	6 (16%)
Severe (>64 mmHg)	3 (8%)
Size of Right Ventricle	
Normal	4 (11%)
Mild Enlargement	7 (18%)
Moderate Enlargement	10 (26%)
Severe Enlargement	17 (45%)
Right Ventricular Dysfunction	
Mild	9 (24%)
Moderate	17 (45%)
Severe	12 (32%)

**Table 3:** Patients’ clinical symptoms, in association with other findings

	**Symptoms**		**P value**
	**Yes (n= 6)**	**No (n= 32)**	
Age (years)	27 ± 4.6	25 ± 9.0	0.533
Sex (F/M)	5/1	8/24	0.012
Age of Prosthesis (years)	1.7 ± 0.8	1.7 ± 1.4	0.635
Malfunction of Prosthetic Valve	5 (83%)	2 (6%)	<0.001
Insufficiency of Prosthesis			0.010
Mild	0	2 (6%)	
Moderate	3 (50%)	3 (9%)	
Moderate to Severe	1 (17%)	1 (3%)	
Right Ventricular Dysfunction			0.518
Mild	2 (33%)	11 (34%)	
Moderate	4 (67%)	13 (41%)	
Severe	0	8 (25%)	
Pulmonary Artery Pressure (mmHg)	26.7 ± 6.8	20.6 ± 8.3	0.081
Peak Pressure Gradient (mmHg)	32.2 ± 24.4	23.1 ± 16.9	0.659
Mean Pressure Gradient (mmHg)	17 ± 12.6	12.8 ± 9.1	0.748
RVOT/PV VTI	0.91 ± 0.01	0.63 ± 0.25	<0.001

The echocardiographic data are summarized in Table 4. The mean peak pressure gradient was 20.2 ± 14.2 mmHg in the normally functioning pulmonary prostheses and 43.9 ± 22.2 mmHg in the malfunctioning pulmonary prostheses. The mean pulmonary artery pressure did not differ between the functioning and malfunctioning valves (21.5 mmHg and 22.1 mmHg respectively).

**Table 4:** Prosthesis malfunction in association with patients’ other findings

	**Malfunction**		**P value**
	**No (n= 31)**	**Yes(n = 7)**		
Age (years)	24.7 ± 9.1	26.9 ± 4.6	0.563
Sex (F/M)	9/22	4/3	0.157
Age of Prosthesis (years)	1.7 ± 1.3	2 ± 1.2	0.544
INR	2 ± 0.4	2.1 ± 0.8	0.292
Right Ventricular Dysfunction			0.085
Mild	9 (29%)	0	
Mild to Moderate	4 (13%)	0	
Moderate	9 (29%)	4 (57%)	
Moderate to Severe	8 (26%)	2 (29%)	
Severe	1 (3%)	1 (14%)	
Insufficiency of Prosthesis			<0.001
Mild	1 (4%)	1 (10%)	
Moderate	0	5 (60%)	
Severe	0	1 (20%)	
Pulmonary Artery Pressure (mmHg)	21.5 ± 8.6	22.1 ± 7.5	0.865
Peak Pressure Gradient (mmHg)	20.2 ± 14.2	43.9 ± 22.2	0.001
Mean Pressure Gradient (mmHg)	11 ± 7	24.3 ± 12.6	0.031

### Patients’ Symptoms

Six patients (16 %) had clinical symptoms of right ventricular failure (fatigue, palpitation or dyspnea). The associations between the symptoms and patients’ characteristics and clinical/echocardiographic findings are presented in Table 3. Female sex was a predictor of symptoms (5 of 6 symptomatic patients and 8 of 32 asymptomatic patients were women, p= 0.012). Five out of the 7 patients with malfunctioning prostheses were symptomatic (p< 0.001). RVOT to PV VTI was 31.5 ± 13.4 in the symptomatic and 0.63 ± 0.25 in the asymptomatic patients (p< 0.001). Prosthetic insufficiency occurred in 4 of 6 symptomatic patients, and 6 of 32 asymptomatic patients (p= 0.01). Our multivariable analysis via a logistic regression model showed that only prosthesis malfunction, prosthesis insufficiency and RVOT/PV VTI had significant associations with the presence of patients’ symptoms (adjusted odds ratio [CI 95%]: 8.69 [1.14–58.82]) .

### Prosthesis Malfunction

Seven (18%) patients had malfunctioning pulmonary prostheses and 2 patients underwent redo PVR. Five year longevity of the pulmonary valve prostheses was 95%. There was no significant association between the severity of RV dysfunction, patient’s age, prostheses valve size and age of the prosthesis in the patients with and without prosthesis malfunction (Table 4). There was a significant association between prosthesis malfunction and prosthesis insufficiency (p< 0.001), increased peak and mean pressure gradient of the prostheses (p values 0.001, and 0.031 respectively). With respect to the 7 malfunctioning prosthetic valves, malfunction occurred in 3 (43%) between the first and the second year, in 2 (29%) between the second and the third year, in 1 (14%) between the third and the fourth year, and in 1 (14%) between the fourth and the fifth year after surgery. There was no significant association between pulmonary prosthesis malfunction and the RV size and function.

The mean PV VTI was significantly greater in the patients with malfunctioning prosthetic valves (p= 0.04). Our logistic regression analysis revealed that only RVOT VTI had a significant association with prosthesis malfunction (adjusted odds ratio [CI 95%]: 1.13 [1.02–1.27]).

## Discussion

There are limited data regarding patients with mechanical pulmonary valve prostheses, with the bulk of the information coming from small sets of patients. The present study shows acceptable midterm outcome of PVR with mechanical prostheses despite the unfavorable reputation of mechanical prosthetic valves in the pulmonary position.^18-20^ In addition, our findings show that thrombosis on mechanical pulmonary prostheses is not a rare complication as was previously mentioned by Haas et al.^21^

In the existing medical literature, there are only a few small studies of patients with mechanical PVR with inconsistent results. Indeed, the reported number of patients with right-sided mechanical prostheses is still too limited and the follow-up duration istoo short to permit any firm conclusions on the safety and durabilityof right-sided mechanical valves.^22-24^

Mechanical valve prostheses carry a lifelong risk of thromboembolicevents even with proper anticoagulation therapy. Mechanical pulmonary prosthesis malfunction occurred in 7 (18%) of our patients. The mean INR in the patients with prosthesis malfunction at the time of admission was 2.1, which denotes inadequate anticoagulation. Most of the cases of prosthesis malfunction (6 out of 7) occurred during the first three years after surgery with an inadequate anticoagulant state, suggesting thrombus formation. Five patients responded well to fibrinolytic therapy based on the guidelines^1^ for thrombosed right-sided prosthetic heart valves (Streptokinase for 24 to 48 hours) with no complications. They subsequently had an uneventful six-month follow-up period.

Miyamura et al.^18^ reported high incidence ofthrombosis in right-sided mechanical valves when he found that two of the five pulmonary valve prostheses (St. Jude Medical) had becomethrombotic despite anticoagulation therapy. The author concluded that the St. Jude medical valve prosthesis was not asuitable prosthesis in the right side of the heart of TOF patients. Waterbolk et al.^24^ in a series of 27 mechanical pulmonaryvalve prostheses demonstrated that the thrombosis of the prosthetic pulmonary valvedid not occur when a proper anticoagulation regimen was maintained which was consistent with Stulak et al. ^25^ study. Accordingly, Haas et al.^21^ reported satisfactory results with the use of a mechanical valveconduit in the pulmonary position at 11 to 63 months’ follow-up, with no evidence of valve failure or tissuegrowth within the valve annulus. All the patients in His study received anticoagulants to maintain an INR of 3.0 to 4.5.

In the current study, there was no significant association between the severity of RV dysfunction, patient’s age, and age of the prosthesis in the patients with prosthesis malfunction. There was a significant association between prosthesis malfunction and increased mean and peak pressure gradient across the prostheses. These data are consistent with those in our previous study on the peak pressure gradient and mean pressure gradient of mechanical pulmonary prostheses.^26^ Interestingly, almost all the patients with prosthesis malfunction had significant prosthesis insufficiency (p< 0.001). In our opinion, new pulmonary prosthesis insufficiency could be a sign of prosthesis malfunction.

## Limitation

The present study does not contain a large number of patients with long-term survival rates and event-free survival rates of the mechanical pulmonary valve prostheses used. Future studies with larger population and long-term follow-up periods of this group of patients are, therefore, required.

## Conclusion

Our study showed acceptable midterm outcome of PVR with mechanical prostheses. Pulmonary prosthesis malfunction occurred in 18% of our patients, mostly as a consequence of thrombus formation. All these cases, however, had an inadequate anticoagulation state.

Thrombosis on mechanical pulmonary valve prostheses may still remain a serious complication. However, with adequate lifelong anticoagulation, theuse of a mechanical valve in the pulmonary position might be considered as an alternative to bioprostheses in patients with severe RV dysfunction or with another mechanical valve.

## Acknowledgments

We thank Pedram Amouzadeh for editorial assistance.

## Ethical issues

Not applicable.

## Competing interests

Authors declare no conflict of interest in this study.
